# Harnessing Computational Approaches for RNA-Targeted Drug Discovery

**DOI:** 10.59566/isrnn.2024.0101001

**Published:** 2024-12

**Authors:** Yuanzhe Zhou, Shi-Jie Chen

**Affiliations:** 1 Department of Physics and Astronomy, University of Missouri, Columbia, MO 65211, USA;; 2 Department of Biochemistry, MU Institute for Data Science and Informatics, University of Missouri, Columbia, MO 65211, USA

**Keywords:** RNA-ligand interaction, RNA-targeted drug discovery, virtual screening, molecular docking, QSAR

## Abstract

RNA molecules have emerged as promising therapeutic targets due to their diverse functional and regulatory roles within cells. Computational modeling in RNA-targeted drug discovery presents a significant opportunity to expedite the discovery of novel small molecule compounds. However, this field encounters unique challenges compared to protein-targeted drug design, primarily due to limited experimental data availability and current models’ inability to adequately address RNA’s conformational flexibility during ligand recognition. Despite these challenges, several studies have successfully identified active RNA-targeting compounds using structure-based approaches or quantitative structure-activity relationship (QSAR) models. This review offers an overview of recent advancements in modeling RNA-small molecule interactions, emphasizing practical applications of computational methods in RNA-targeted drug discovery. Additionally, we survey existing databases that catalog nucleic acid-small molecule interactions. As interest in RNA-small molecule interactions grows and curated databases expand, the field anticipates rapid development. Novel computational models are poised to enhance the identification of potent and selective small-molecule modulators for therapeutic needs.

## INTRODUCTION

Recent years have seen growing interest in RNA-targeted drug discovery due to RNA’s diverse functional and regulatory roles in gene expression, protein synthesis, and viral replication^1–12^. Identifying small molecules that bind to specific RNA targets could lead to novel therapeutic agents for treating diseases associated with dysregulated RNA functions. Compared to the protein-targeted approaches, targeting RNA significantly broadens the druggable genome and may provide more effective disease treatments due to RNA’s critical roles in gene expression and regulation^2, 3, 6, 13, 14^. It is estimated that ~85% of the human genome is transcribed into RNAs^2, 15^, while only approximately 1.5% encodes proteins^3, 6, 13, 14, 16–20^. The vast majority of the transcribed RNAs are non-coding RNAs (ncRNAs), which do not encode proteins but rather serve crucial enzymatic, structural, and regulatory functions in gene expression. Recent studies have shown that many ncRNAs, including microRNAs (miRNAs), long non-coding RNAs (lncRNAs), and circular RNAs (circRNAs), have been found to be associated with human diseases such as cancer and neurodegenerative disorders, making them potential new drug targets^21–26^. The discovery of ribocil and its derivatives^27–30^ - potent synthetic ligands that bind to FMN riboswitches with chemically distinct scaffold compared to the natural ligand - demonstrates the possibility of using riboswitches as the antibacterial drug targets^31^, alternative to the traditional ribosomal RNAs (rRNAs) targets^32, 33^, to combat bacterial infections. Other RNA targets, such as survival of motor neuron 2 (SMN2) pre-mRNA in spinal muscular atrophy (SMA)^34^, ribosomal frameshifting stimulation element (FSE) within the severe acute respiratory syndrome coronavirus 1/2 (SARS- CoV-1/2) RNA genome^35–39^, element for nuclear expression (ENE) triple helixes presented in Kaposi’s sarcoma-associated herpesvirus (KSHV) polyadenylated nuclear (PAN) lncRNA [40], human immunodeficiency virus type 1 (HIV-1) trans-activation response (TAR) element^41, 42^, a flexible “priming loop” region of the *cis*-acting regulatory element (ϵ) located at the 5’-ends of the hepatitis B virus (HBV) pre-genomic RNA (pgRNA)^43, 44^, have also been shown to be targetable by small molecule compounds. Notably, FDA’s approval of an RNA splicing modulator, Evrysdi (risdiplam) in August 2020 for treating SMA represents the first small molecule drug designed to directly target non-ribosomal RNA, paving the way for further advancements in RNA-targeted drug discovery^11, 45–47^.

**Fig. 1.**
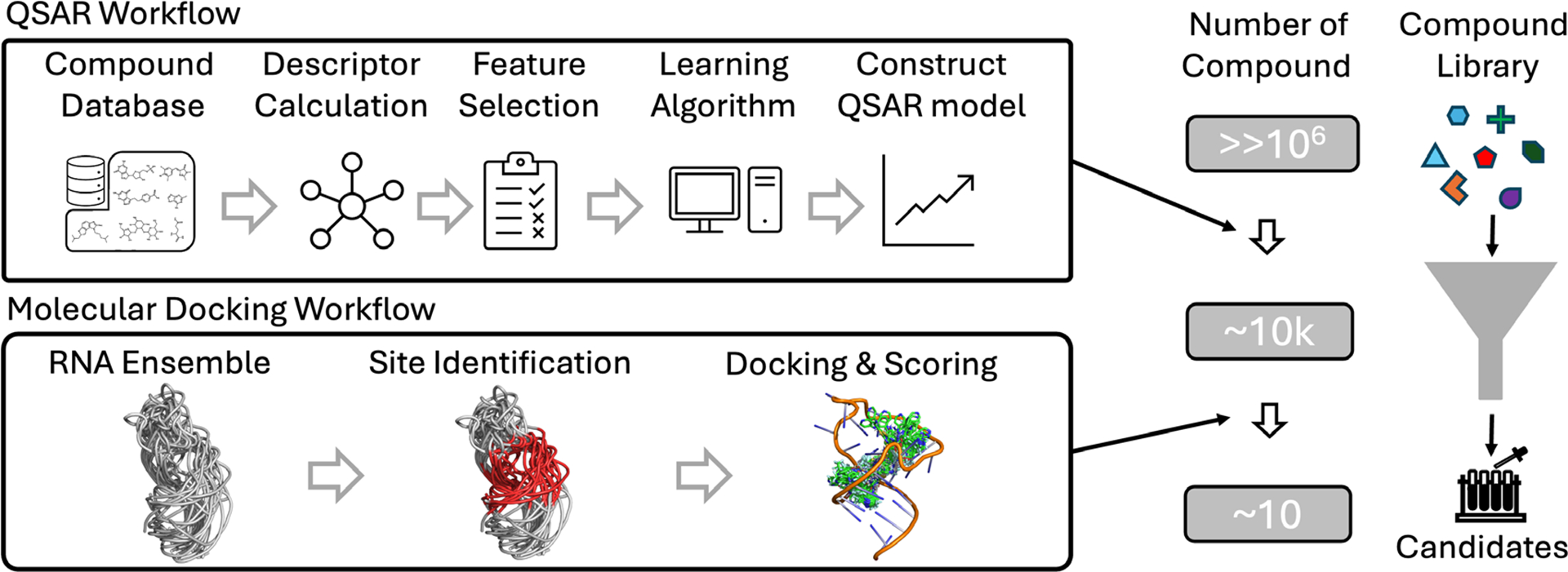
Examples of QSAR and molecular docking workflows for RNA-targeted virtual screening (VS). The illustrated VS combines QSAR and molecular docking in a consecutive manner, effectively reducing computational costs while retaining similar screening power.

Despite the growing interest in RNA-targeted drug discovery, computational modeling of RNA-small molecule interactions is still in its infancy. The *in silico* process of identifying lead compounds for specific RNA targets varies based on the availability of RNA structural information and computational resources, ranging from quantitative structure-activity relationship (QSAR)-based approaches to structure-based approaches. QSAR-based approaches^48–51^ build simple analytic models to establish the relationships between a set of predefined molecular descriptors (e.g., physicochemical properties such as molecular weight, polarizability, surface area, number of H-bond donors/acceptors, and 3D shape) and binding-related quantities for experimentally identified bound ligands. These QSAR models can then be used to quantify RNA-small molecule associations for given small molecule compound libraries. In contrast, structure-based approaches^52–65^, such as molecular docking, leverage prior knowledge of RNA at various structural levels to gain detailed insights into drug-target interaction mechanisms. These methods provide a more comprehensive understanding of the binding process but typically require more extensive computational resources and structural data. See Fig. 1 for examples of QSAR and molecular docking workflows.

However, RNA flexibility and data scarcity pose significant challenges in RNA-targeted drug discovery. Unlike proteins, RNA molecules are highly dynamic, adopting multiple, rapidly inter-converting conformations^1, 14, 66–68^. This inherent flexibility complicates the design of small molecules that can reliably bind to specific RNA structures. Recent comparative studies have shown that current docking software struggles to model the conformational changes of RNA receptors induced by ligand binding^69, 70^. Accurate modeling of RNA-ligand interactions requires knowledge of RNA conformational ensemble. Furthermore, the limited availability of high-quality structural data on RNA-small molecule complexes further complicates the drug design process, especially for data-driven approaches involving machine learning (ML)^71, 72^. In the following sections, we will focus on available database resources and practical applications of computational tools that facilitate the early stages of RNA-targeted small molecule drug discovery. We will not delve into the detailed methodologies employed by various computational models, as these have been covered in comprehensive reviews elsewhere^66, 71, 73, 74^.

## DATABASES WITH KNOWN RNA-LIGAND INTERACTIONS

RNA-targeted small molecule drug discovery is still in its early stage. The development of robust predictive models for RNA-small molecule interactions is currently hindered by the limited availability of high-quality, experimentally validated data. While several databases have emerged in recent years, many lack substantial amount of 3D structural information for RNA-small molecule systems. Data-driven models, particularly deep learning (DL) models — a subset of ML models that leverages deep neural networks to perform complex tasks — often comprise a significant number of trainable parameters. These models require sufficient high-quality training data to mitigate common issues such as overfitting. A notable example is AlphaFold 3^75^, which can model interactions between diverse biomolecules, including RNA-small molecule interactions. To further improve current DL models, there is a pressing demand for carefully curated benchmark datasets that encompass detailed 3D structural information and experimentally measured properties, such as binding affinity. For example, in the most recent version of PDBbind (version 2021), there are 22,920 protein-ligand complexes with experimentally measured binding affinity data compared to only 171 nucleic acid-ligand complexes with affinity data^76^. By offering a standardized training and testing dataset, a well-curated database of RNA-small molecule interactions would be an invaluable resource for the scientific community, providing a solid foundation for advancing various computational approaches to understanding RNA biology and RNA-targeted therapeutics. In the following, we briefly overview currently available databases containing curated nucleic acid-small molecule interactions published after 2016. See Table 1 for a list of reviewed databases.

### Databases without 2D/3D structural information of nucleic acid

The Nucleic Acid Ligand Database (NALDB)^77^ is a database providing detailed experimental information on 3,610 ligands that target several types of nucleic acid structures. The information includes target sequence, ligand structures and chemical properties, along with their K_i_, K_D_, and IC_50_ values, etc. The ligands are broadly categorized into six classes based on their corresponding binding nucleic acid partners: double-stranded DNA/RNA, G-quadruplex DNA/RNA, DNA/RNA aptamers, and special DNA/RNA structures. The NALDB web portal offers search functionality that enables users to filter the database based on drug-like properties or similarities to user-uploaded query molecules.

NoncoRNA^80^ is a manually curated database providing experimental information on interactions between various ncRNAs and drugs. It contains 8,233 entries involving 5,568 ncRNAs and 154 drugs across 134 cancer types. The database offers a web interface for browsing, searching, and downloading the data. Each entry in NoncoRNA contains detailed information on the ncRNAs, drugs, and related cancers, including ncRNA expression patterns, experimental detection techniques, drug responses, and literature references, etc.

To investigate the druggability of RNAs with small molecules, Rizvi *et al*.^5^ employed the automated ligand identification system (ALIS) affinity-selection mass spectrometry platform to screen 42 RNAs across diverse RNA classes against approximately 60,000 compounds. This screening generated millions of target-compound interaction data points. From the identified RNA binders, the researchers constructed an RNA-focused library of roughly 3,700 small molecules with enhanced RNA-targeting capabilities. Several RNA targets in the database can be accessed by modifying existing experimentally solved 3D structures in the RCSB PDB database^89^. Unfortunately, the authors did not provide public access to the detailed screening results.

Recently, Yazdani *et al*.^50^ reported the Repository of BInders to Nucleic acids (ROBIN), which contains small molecule microarray (SMM) screening results for 36 individual nucleic acids (27 RNA and 9 DNA) against a library of 24,572 small molecules. The database identified 2,003 RNA-binding small molecules targeting various RNA motifs, including RNA hairpins, DNA/RNA G-quadruplexes, RNA triple helices, RNA pseudoknots, and RNA three-way junctions. Notably, ROBIN is currently the largest public database for nucleic acid-small molecule interactions. Although lacking RNA 3D structure information, many RNA targets in ROBIN can be obtained by modifying related experimentally solved 3D structures available in the RCSB PDB database^89^, making it a valuable benchmark dataset for developing effective nucleic acid-specific virtual screening tools.

### Databases with 2D/3D structural information of nucleic acid

Inforna 2.0^78^ is a template database that contains 1,936 pairs of known RNA secondary structure motif-ligand bound complexes, comprising 244 unique small molecules and 1,331 RNA motifs. The data was collected from an in-house two-dimensional combinatorial screen against a panel of RNA secondary structures, including hair-pins, symmetric and asymmetric internal loops, and bulges, etc. The database can identify RNA structural motifs similar to the user-provided RNA secondary structures and perform similarity searches in large compound libraries using the native bound ligands as lead compounds.

RNALigands^83^ is a database focused on collecting RNA secondary structural motifs and their bound small molecule ligands. It contains 841 RNA-ligand pairs gathered from three sources: RCSB PDB database (386 pairs)^89^, R-BIND (67 pairs)^90^, and Inforna 2.0 (388 pairs)^78^. By integrating a motif alignment algorithm, RNALigands can identify secondary structure motifs from user input that are similar to those stored in the database. The matched RNA motifs and their bound small molecule ligands can provide valuable information for identifying lead compounds.

RNA-targeted BIoactive ligaNd Database (R-BIND) 2.0^84^ is the biennial update of the original R-BIND database^90^, designed to facilitate the discovery of RNA-targeted chemical probes. It contains comprehensive information on reported chemical probes that target non-rRNA and exhibit biological activity. The database includes 1,494 recorded RNA-small molecule interactions for 153 unique small molecules, where experimental details for *in vitro* assays, cell-based assays, and/or animal model studies conducted after the primary screen for each ligand can be found. The web portal of the database also offers search functionality based on RNA structure. The algorithm uses an input RNA sequence (or structure of interest), conducts RNA structure prediction, and then returns ligands in the database that bind that motif (if any) based on RNA secondary structure and size.

Several other databases also contain 3D structural information for nucleic acid-small molecule complexes. PDBbind v. 2021^76^ database contains 171 nucleic acid-ligand complexes with solved structures and measured binding affinities. RNA-Small molecule Interaction Miner (R-SIM)^86^ database includes 2,501 experimentally validated RNA-small molecule interactions, covering 461 unique RNA targets and 1,288 unique small molecules. HARIBOSS (Harnessing RIBOnucleic acid- Small molecules Structures)^85^ database comprises 311 compounds, 862 RNA-small molecule complexes, and 1471 pockets collected from RCSB PDB database^89^.

### Databases focusing on small molecules targeting specific nucleic acid types

Currently, several databases have been curated for small molecules interacting with specific nucleic acid types, including riboswitches^79, 82, 87^, nuclear acid aptamers^88^, and nucleic acids with special topologies (e.g., G-quadruplex and i-Motif^81^). Three databases focus on riboswitches are RiboD^79^, RSwitch^82^, and Ribocentre-switch^87^. Among these, Ribocentre-switch is the most recent and comprehensive database encompassing 56 riboswitches and 26 orphan riboswitches from over 430 references, with a total of 89,591 sequences. It provides information on target sequences, structures, functions, ligand binding pockets, and biological applications. AptaDB^88^ is an aptamer database derived from experimentally validated aptamer-target interaction data collected from the literature. It includes information such as aptamer structure and experimental binding affinities. Currently, AptaDB contains 1,350 experimentally validated aptamer-target interactions, of which 393 involve small molecules. G4LDB 2.2^81^ focuses on nucleic acid structures with special topologies, such as G-quadruplex and i-Motif. It currently contains more than 3,200 G-quadruplex/i-Motif ligands, with ~28,500 activity entries.

## COMPUTATIONAL MODELING OF RNA-SMALL MOLECULE INTERACTIONS AND THEIR APPLICATIONS IN RNA-TARGETED DRUG DISCOVERY

Over the years, researchers have developed various computational models to streamline the early stages of RNA-targeted drug development. These models aim to reduce the time and cost associated with experimental testing by facilitating critical processes such as binding site detection, binding mode prediction, and ultimately, lead identification and optimization^66, 71–74^. See Table 2 for a list of applications reviewed here for RNA-targeted drug discovery.

### Structure-based virtual screening identifies RNA-targeting small molecules

Structure-based approaches, such as RNA-ligand molecular docking, provide detailed insights into binding modes and the structural basis of RNA-small molecule recognition, crucial for designing potent and selective drugs. Unlike protein-ligand docking, software specifically designed for RNA-ligand interactions is limited^66^. Avaliable tools include all-in-one docking software (AutoDock^52^, DOCK 6^53^, rDock^54^, RLDOCK^55, 56^, NLDOCK^57^) and standalone scoring functions ranging from physics-based to knowledge-based and machine-learning models (DrugScoreRNA^58^, LigandRNA^59^, SPA-LN^60^, ITScore-NL^61^, AnnapuRNA^62^, RNAPosers^63^, RNAmigos^65^, and RNAPosers-ssMD^64^). While significant progress has been made in predicting the binding of a flexible ligand and a rigid *holo*-form RNA structure with limited side chain flexibility^66^, capturing the ligand-modulated RNA conformational dynamics remains challenging. Small molecule recognition is sensitive to local binding pocket conformations, and even minor deviations from the *holo*-conformation could render current docking methods ineffective. This issue is far more pronounced for RNA than protein, as RNA ligand binding site conformations are more flexible^1, 14, 91^. Most current molecular docking studies employ an ensemble-based virtual screening (EBVS) workflow to address this issue. In this approach, a candidate compound library is docked into an ensemble of RNA structures rather than a single rigid conformation. This structural ensemble can be obtained through either computational method, such as MD simulation, or experiments data, such as those from NMR residual dipolar coupling (RDC). However, the EBVS approach is typically more computationally expensive and may not provide significant improvements over single-conformation docking without experimentally informed structural information. Consequently, only a limited number of successful molecular docking-based virtual screenings against RNA targets have been reported to date^35–37, 41–44, 92, 93^.

SARS-CoV-1 and SARS-CoV-2 are two coronavirus strains responsible for the SARS outbreak and COVID-19 outbreak, respectively^94^. These viruses utilize ribosomal frameshifting (RF), also known as translational frameshifting, a conserved mechanism that allows multiple proteins to be produced from a single mRNA sequence^95^. During translation, RF occurs when the ribosome shifts its reading frame, typically by one or two nucleotides, resulting in the synthesis of a protein different from what the standard reading frame would produce. This process enables efficient use of genetic material and is crucial for viral replication and gene expression regulation. Studies have shown that the RNA pseudoknot at the −1 RF site effectively stimulates −1 RF in SARS-CoV-1^35, 36^. Disrupting −1 RF by targeting this pseudoknot with a small molecule could potentially inhibit viral infectivity and production. Park *et al*.^37^ explored this possibility by using DOCK 4.0^96^ to screen 80,000 compounds against a computationally predicted and optimized SARS-pseudoknot structure. The structure was initially predicted using PSEUDOVIEWER^97, 98^ and the SYBYL 6.9 system (Tripos, Inc.), then optimized with AMBER 8^99^. Based on DOCK scoring function and visual inspection, 58 highly ranked compounds were selected for further evaluation. *In vitro* and *cell-based* −1 RF assays on these compounds led to the identification of a novel ligand capable of dramatically inhibiting SARS-CoV-1’s −1 RF. Recently, Mathez *et al*.^100^ employed a similar molecular docking-based approach using the Schrodinger Suite version 2023–2^101^ to virtually screen the ROBIN dataset^50^, where 2,003 RNA-targeting compounds were docked against an ensemble of MD-refined crystal structures of the pseudoknot involved in the −1 RF. After performing high-throughput, standard precision (SP), and extra precision (XP) docking, the final poses of each compound were refined using Prime MM-GBSA. Twenty-one molecules binding to at least 3 clusters were visually inspected and experimentally validated against the wild-type virus. Several compounds demonstrated antiviral activity with varying EC_50_ values, and one compound induced a significant reduction in frameshift efficiency. Further investigation into analogues of this compound revealed one analogue with more potent antiviral activity and an improved ability to reduce frameshift efficiency.

Riboswitches are naturally occurring RNA segments located in the untranslated region (UTR) of messenger RNA (mRNA) molecules. They contain a ligand-binding aptamer domain where specific ligands can bind and induce alternative folding of the downstream expression platform, regulating transcription, translation, or other gene expression process^102, 103^. With over 55 distinct classes of natural riboswitches discovered that selectively sense small molecules or elemental ions, and many more predicted to exist^102, 103^, riboswitches present an alternative target for antibacterial drug development. This approach could help combat emerging multidrug resistant bacteria, complementing traditional rRNAs targeting strategy^31–33^. Daldrop *et al*.^92^ utilized DOCK software to screen 2,615 unique compounds (including eight experimentally verified hits) against the adenine riboswitch (AR) and a related guanine riboswitch carrying a point mutation (GRA). Using a force-field-based scoring function modified with RNA-specific parameters, Daldrop *et al*. achieved an area under the receiver operation characteristic curve (AUROC) of 0.98. Further molecular docking and experimental investigation of top-ranked commercially available compounds identified four hits that bind to GRA, with two exhibiting novel scaffolds compared to the known ligands [92]. More recently, Kallert *et al*.^93^ investigated the capabilities of protein-based docking programs to reproduce native binding modes and discriminate known binders from decoys. Kallert *et al*. conducted structure-based virtual screening by docking 12,507 compounds into the *Bacillus subtilis* preQ_1_ riboswitch aptamer domain using protein-ligand docking software FRED^104^, HYBRID^105^, and FlexX^106^. Subsequent microscale thermophoresis assays identified six active compounds out of 23 tested VS hits, with K_D_ values ranging from 29.5 nM to 11.0 μM. Notably, the VS results indicated that combining docking score-based ranking and pharmacophore hypothesis-based binding mode interpretation (i.e., human expert intervention) is superior to the docking score-based ranking alone, providing guidance for hit selection.

HIV, a subgroup of retrovirus, can cause acquired immunodeficiency syndrome (AIDS) after infection. Of the two types of HIV (i.e., HIV-1/2), HIV-1 is the one that was first discovered and is the more dominant and pathogenic strain^107^. Studies have shown that the trans-activation response (TAR) element of HIV-1, a well folded stem-bulge-loop RNA in the 5’ UTR of the viral genome, is essential for HIV replication. It interacts with the Tat protein, helping recruit host transcriptional machinery to facilitate efficient viral DNA transcription^108^. This central role underscores TAR’s potential as a therapeutic target. To account for TAR’s large conformational flexibility upon ligand binding, Stelzer *et al*.^41^ adopted an EBVS approach. Stelzer *et al*. used structural data, such as NMR residual dipolar coupling, to construct an RNA dynamic ensemble for molecular docking studies. Using the Internal Coordinate Mechanics (ICM, Molsoft^109^) docking program, They screened ~51,000 small molecules against each of the 20 conformers in the TAR ensemble. Fluorescence-based assays of the top 57 commercially available hits experimentally validated six active small molecules that bind TAR and inhibit its interaction with Tat, with K_D_ and K_i_ values ranging from 55 nM to 122 μM and from 710 nM to 169 μM, respectively. Ganser *et al*.^42^ employed a similar approach to validate ability of EBVS to discriminate between known nonhits and hits targeting TAR. Ganser *et al*. docked ~100,000 drug-like molecules, including 177 experimentally verified hits, against the RDC-informed TAR conformational ensemble^110^ using ICM^109^. EBVS enriched the compound library with an AUROC of 0.88 and identified 42% of hits after screening only 2% of the compounds, which corresponds to an enrichment factor of 21. By varying ensemble size and using TAR ensemble generated computationally without considering experimental structural information, the authors observed a significant decrease in enrichment performance demonstrating the importance of an accurate, large RNA conformational ensemble.

HBV infection causes liver disease and accounts for over 600,000 deaths annually^111^. Recent experimental studies by LeBlanc *et al*.^43^ identified several small molecules, including Raloxifene, Bazedoxifene, and a *de novo* derivative, that selectively target a flexible “priming loop” region of the *cis*-acting regulatory element (ϵ) located at the 5’ ends of the HBV pre-genomic RNA (pgRNA). To further investigate the potential of HBV ϵ as a promising drug target to complement current anti-HBV therapies^43^, Olenginski *et al*.^44^ carried out virtual screening against HBV ϵ. Olenginski *et al*. docked 1,604 FDA-approved compounds into NMR-solved RNA ensemble structures with AutoDock Vina^112^. Using the predicted affinity of the known ϵ-targeting ligand Raloxifene as a cutoff, 66 top-ranked compounds were selected based on commercial availability and potential adverse effects. Among these, three anti-Hepatitis C virus (HCV) compounds (Simeprevir, Ledipasvir, and Daclatasvir) were found to bind with full-length HBV ϵ, showing approximate EC_50_ values of 298, 145, and 62 μM, respectively. Further dye-displacement assays with additional RNAs containing structural elements similar to HBV ϵ validated Daclatasvir as a selective ϵ-targeting compound^44^. This work supports the possibility that targeting ϵ dynamics may be an effective anti-HBV therapeutic strategy.

While molecular docking is widely used in structure-based virtual screening, approaches solely relying on 2D structural information have also found successfully applications in RNA-targeted drug discovery. One such model is Inforna^113^, a template-based method capable of identifying hit compounds for given RNA target based on their similarities to the known compound-interacting RNA secondary structure motifs such as hairpins, symmetric and asymmetric internal loops, and bulges. The latest iteration, Inforna 2.0^78^, contains 1,936 pairs of known RNA secondary structure motif-ligand complexes. For a given RNA target, Inforna first identifies its secondary structure motifs and matches them against the template database. For any matching motif, it then finds the corresponding ligand partners and calculates fitness scores. These scores provide a measure of RNA-ligand binding affinity as well as the selectivity of the RNA motif against other small molecules. RNAs with high selectivity and affinity fitness scores are more likely to be druggable. Using the top-scored small molecules as lead compounds, chemical similarity screening against compound libraries can identify potential potent binders. Compared to traditional molecular docking-based approaches, Inforna and Inforna 2.0 take advantage of known interactions between small molecules and RNA structural motifs. The similarity-based approach can provide a massive speedup when performing large-scale compound screening. Additionally, Inforna and Inforna 2.0 do not require 3D structural information of the target, which makes them suitable for tasks where only 2D structural information is available. In practice, Inforna and Inforna 2.0 can complement molecular docking, where one can first use Inforna and Inforna 2.0 to reduce the compound library to a reasonable size and then proceed with molecule docking that provides a refined compound ranking and reveals details of the interaction mechanism. Inforna has proven successful in various studies^6, 114^, such as identifying small molecules that target oncogenic miRNA precursors^115, 116^, an A bulge in the iron responsive element (IRE)^117^ of the SNCA mRNA related to Parkinson’s disease^118^, and a miRNA-200 family member precursor associated with type 2 diabetes^119^, etc.

### Quantitative Structure-Activity Relationship (QSAR) models for RNA-small molecule binding

Studies employing QSAR models have demonstrated their effectiveness in prioritizing drug candidates and optimizing lead compounds^47–51, 72, 120–123^. Compared to structure-based approaches, QSAR models are computationally efficient, allowing researchers to screen large compound libraries. These models leverage statistical and machine learning techniques to analyze existing data on known compounds, identifying patterns that link structural characteristics to specific biological effects or physicochemical properties such as binding affinity. However, QSAR models have limitations. They are typically trained on small molecule datasets for specific RNA targets, which makes them difficult to generalize to other RNA types. Additionally, they usually cannot provide detailed descriptions of the microscopic interactions involved in binding. Despite QSAR’s success in protein-based drug design, only a few QSAR studies have been conducted for RNA targets. In the following paragraph, we discuss several selected applications of QSAR in RNA-targeted drug discovery. Interested readers are directed to the references^47–51, 72, 120–123^ in addition to those reviewed in the main text.

Cai *et al*.^49^ developed a workflow utilizing QSAR to connect molecular descriptors of a given ligand with its binding profiles, including binding affinity K_D_ and kinetic rate constants (k_on_ and k_off_), against a specific RNA. To test the proposed workflow, the authors chose the HIV-1 TAR element as the RNA target, since TAR is a well-studied antiviral target with many experimentally verified bound compound candidates for training^108, 124–126^. The training set comprised 48 compounds, including 29 reported TAR-binding ligands and 19 compounds with known RNA targeted scaffolds. For each ligand, 435 calculated descriptors, ranging from topological to electrostatic terms, along with their measured binding parameters, were used to train the regression model. The least absolute shrinkage and selection operator (LASSO) was employed prior to training to select an optimal set of molecular descriptors. Comparison of predictive performance on the test set between multiple linear regression (MLR) model and various ensemble tree methods (e.g., random forest and boosting trees) showed that MLR gave overall best correlation between predicted and measured binding parameters. One possible reason is the utilization of the LASSO for descriptor selection prior to MLR. Lasso has been widely used in QSAR studies to control model complexity by applying a penalty constraint to the loss function during modeling. This helps identify the most relevant descriptors and reduce noise, which might have been particularly effective for this dataset.

A common practice after identifying lead compounds involves optimization cycles where certain chemical functional groups are replaced to improve the potency of the candidates. With the availability of large virtual screening libraries of small molecules and experimentally validated activities, the principles governing ligand binding can be learned by machine learning models to guide drug design. Grimberg *et al*.^51^ compared several machine learning-based models, including LASSO regression, decision tree classifier, and convolutional neural network (CNN), for optimizing phenylthiazole-containing small-molecule inhibitors targeting the RNA hairpin 91 within the ribosomal peptidyl transferase center (PTC) of *Mycobacterium tuberculosis*. By training machine learning models on a dataset containing 791 drug-sized molecules with the 2-phenylthiazole moiety and their predicted binding scores for the RNA target (generated by the molecular docking program AutoDock^52^), chemical features important for small molecule binding were identified and subsequently used as guidelines for synthesizing new inhibitors. Functional validation was conducted after synthesizing ten small molecules, of which four were found to be potent inhibitors that target hairpin 91 in the ribosomal PTC of *M. tuberculosis* and stop translation.

## CONCLUSIONS

RNA-targeted drug discovery is a rapidly evolving field with immense potential for addressing various human pathologies, from bacterial and viral infections to cancer. Although structure-based approaches such as molecular docking and QSAR modeling have already demonstrated success in identifying novel RNA-targeting compounds^6, 37, 41, 42, 44, 51, 92, 93, 115, 117^, significant challenges still remain, particularly in accounting for RNA’s inherent flexibility and the relative scarcity of high-quality structural and binding data. The development of more advanced molecular tools for RNA-based therapeutics^127–129^, together with more comprehensive and well-curated databases of RNA-small molecule interactions, as discussed in this review, will be crucial for advancing the field. These resources will provide the necessary data to train more sophisticated machine learning models and improve our understanding of the principles governing RNA-ligand recognition. With major pharma companies converging upon this field and forging various biotech-pharma alliances^11^, we anticipate an unprecedented increase in the demand for efficient and accurate computational methods for modeling RNA-small molecule interactions. This may include the development of RNA-specific scoring functions, improved modeling of RNA dynamics upon ligand recognition, and integration of experimental data derived from NMR, X-ray crystallography, and cryo-EM to generate more accurate structural ensembles. Additionally, the application of deep learning approaches is likely to yield powerful new predictive models as more data becomes available. As computational methods for RNA-small molecule binding continue to advance, we can realistically anticipate a notable rise in RNA-targeted small molecule therapeutics entering clinical trials in the coming years.

## Figures and Tables

**Table 1.** Databases containing RNA-small molecule interactions published after 2016

Name	Year	NA Type	# of NA-SM Interactions	Focus and Strength	Web Portal	Ref
NALDB	2016	RNA/DNA	3,610	SM-NA association (contains K_*i*_, K_*D*_, IC_50_)	Yes	77
Inforna 2.0	2016	RNA	1,936	SM-RNA association (contains RNA 2D info)	Yes^c^	78
RiboD	2019	RS	1,907^a^	information of RS and gene/operon it regulates	Yes	79
NoncoRNA	2020	ncRNA	8,233	ncRNAs and drug associations	Yes	80
Rizvi *et al*.	2020	RNA	~2,300,000^b^	SM-RNA association (large-scale screening)	No	5
G4LDB 2.2	2021	G4/iM	28,500	SM-G4/iM association	Yes	81
RSwitch	2021	RS	215^a^	information of pathogenic bacterial RS	Yes	82
PDBbind v.2021	2021	RNA/DNA	171	SM-NA association (contains 3D structures and binding properties)	Yes	76
RNALigands	2022	RNA	841	SM-RNA association (contains RNA 2D info)	Yes	83
R-BIND 2.0	2022	RNA	1,494	SM-RNA association (contains RNA 2D info)	Yes	84
HARIBOSS	2022	RNA	1,471	SM-RNA association (contains 3D structures)	Yes	85
R-SIM	2023	RNA	2,501	SM-RNA binding affinities	Yes	86
ROBIN	2023	RNA/DNA	1,627,072^b^	SM-NA association (large-scale screening; publicly available)	No^d^	50
Ribocentre-switch	2023	RS	89,591^a^	information of RS and related ligand binding	Yes	87
AptaDB	2024	RNA/DNA	393	information of NA aptamers from SELEX-based screening	Yes	88

a Number of riboswitch sequences.

b Distinct from other studies, the full results of probed binding interactions were reported, including binders and non-binders.

c Available upon request.

d No official website but the dataset is downloadable through the publisher’s website.

NA, nucleic acid; SM, small molecule; RS, riboswitch; ncRNA, non-coding RNA; G4, G-quadruplex; iM, i-Motif; SELEX, systematic evolution of ligands by exponential enrichment.

**Table 2.** Applications of RNA-targeted drug discovery

RNA Target	Year	Computational Method	Ref
SARS-CoV-1 RF	2011	Docking	[37]
Purine RS	2011	Docking	[92]
HIV-1 TAR	2011/2018	Docking	[41, 42]
PreQ_1_ RS	2022	Docking	[93]
HBV ϵ	2023	Docking	[44]
SARS-CoV-2 RF	2024	Docking	[100]
Inforna	2017/2020	Template-based scoring	[115, 117, 119]
RNA hairpin^a^	2022	QSAR	[51]

a Hairpin 91 within ribosomal peptidyl transferase center (PTC) of *Mycobacterium tuberculosis*.

RF, ribosomal frameshifting; RS, riboswitch; TAR, trans-activation response.

## Data Availability

The data supporting the findings of this study are available in the manuscript or in the supplementary materials.

## ABBREVIATIONS:

ncRNAs: non-coding RNAs
miRNAs: microRNAs
lncRNAs: long non-coding RNAs
circRNAs: circular RNAs
rRNAs: ribosomal RNAs
SMN2: survival of motor neuron 2
SMA: spinal muscular atrophy
FSE: frameshifting stimulation element
SARS-CoV: severe acute respiratory syndrome coronavirus
ENE: element for nuclear expression
KSHV: Kaposi’s sarcoma-associated herpesvirus
PAN: polyadenylated nuclear
HIV-1: human immunodeficiency virus type 1
TAR: trans-activation response
HBV: hepatitis B virus
pgRNA: pre-genomic RNA
QSAR: quantitative structure-activity relationship
ML: machine learning
VS: virtual screening
DL: deep learning
NA: nucleic acid
SM: small molecule
RS: riboswitch
G4: G-quadruplex
iM: i-Motif
SELEX: systematic evolution of ligands by exponential enrichment
ALIS: automated ligand identification system
SMM: small molecule microarray
EBVS: ensemble-based virtual screening
RDC: residual dipolar coupling
RF: ribosomal frameshifting
UTR: untranslated region
mRNA: messenger RNA
AUROC: area under the ROC curve
AIDS: acquired immunodeficiency syndrome
HCV: hepatitis C virus
IRE: iron responsive element
LASSO: least absolute shrinkage and selection operator
MLR: multiple linear regression
CNN: convolutional neural network
PTC: peptidyl transferase center
